# An mHealth Platform for Augmenting Behavioral Health in Primary Care: Longitudinal Feasibility Study

**DOI:** 10.2196/36021

**Published:** 2022-07-01

**Authors:** Khatiya Chelidze Moon, Michael Sobolev, Megan Grella, George Alvarado, Manish Sapra, Trever Ball

**Affiliations:** 1 Zucker Hillside Hospital Glen Oaks, NY United States; 2 Cornell Tech New York, NY United States; 3 Northwell Health Manhassett, NY United States

**Keywords:** collaborative care, mobile health, psychiatry, depression, virtual care, psychoeducation, mobile app, mobile phone

## Abstract

**Background:**

The collaborative care model is a well-established system of behavioral health care within primary care settings. There is potential for mobile health (mHealth) technology to augment collaborative behavioral health care in primary care settings, thereby improving scalability, efficiency, and clinical outcomes.

**Objective:**

We aimed to assess the feasibility of engaging with and the preliminary clinical outcomes of an mHealth platform that was used to augment an existing collaborative care program in primary care settings.

**Methods:**

We performed a longitudinal, single-arm feasibility study of an mHealth platform that was used to augment collaborative care. A total of 3 behavioral health care managers, who were responsible for coordinating disease management in 6 primary care practices, encouraged participants to use a mobile app to augment the collaborative model of behavioral health care. The mHealth platform’s functions included asynchronous chats with the behavioral health care managers, depression self-report assessments, and psychoeducational content. The primary outcome was the feasibility of engagement, which was based on the number and type of participant-generated actions that were completed in the app. The primary clinical end point was a comparison of the baseline and final assessments of the Patient Health Questionnaire-9.

**Results:**

Of the 245 individuals who were referred by their primary care provider for behavioral health services, 89 (36.3%) consented to app-augmented behavioral health care. Only 12% (11/89) never engaged with the app during the study period. Across all participants, we observed a median engagement of 7 (IQR 12; mean 10.4; range 0-130) actions in the app (participants: n=78). The chat function was the most popular, followed by psychoeducational content and assessments. The subgroup analysis revealed no significant differences in app usage by age (*P*=.42) or sex (*P*=.84). The clinical improvement rate in our sample was 73% (32/44), although follow-up assessments were only available for 49% (44/89) of participants.

**Conclusions:**

Our preliminary findings indicate the moderate feasibility of using mHealth technology to augment behavioral health care in primary care settings. The results of this study are applicable to improving the design and implementation of mobile apps in collaborative care.

## Introduction

### Background

The reach of behavioral health services is insufficient for meeting the needs of the population [1,2]. The collaborative care model (CoCM) is a framework that attempts to meet this vast need for behavioral health services by embedding these services in primary care settings [3]. The CoCM is a system of outcome-driven, stepwise care for systematically identifying individuals who would benefit from behavioral health treatment and supporting primary care clinicians in their management. The model has been adapted by many health systems since its introduction in the 1990s and is considered best practice [4-6]. Unfortunately, there are challenges that limit the scalability of the CoCM, including financial and operational barriers [7,8]. Innovative approaches are needed to support these existing models of behavioral health care [9].

There is emerging evidence that digital and mobile health (mHealth) technologies have the potential to improve the reach of the CoCM [9-12]. Given the near ubiquity of smartphones with app capabilities, health systems are increasingly interested in understanding whether these tools can be harnessed to further extend collaborative care [13,14]. There are several meaningful ways that mobile apps could be used to augment collaborative care. The CoCM relies strongly on a measurement-based system of care in which validated clinical assessments (eg, the Patient Health Questionnaire-9 [PHQ-9]) are regularly collected to assess clinical responses [15]. mHealth platforms could help decrease care providers’ workload by automating the collection of these measures [12,13]. Apps could also support clinical decision-making by collecting clinical information more frequently than what is currently possible [10]. Moreover, apps could facilitate more frequent communication between patients and care providers [16,17]. Finally, apps can act as repositories for educational materials and self-guided modules for reinforcing concepts that are learned in therapy and promoting patient engagement [13,18-20]. All of these factors have the potential to increase patient engagement in care and, eventually, result in improved clinical outcomes.

Despite the theoretical benefits of app-augmented collaborative care, relatively little is still known about the feasibility of app usage in collaborative care. It is unknown whether patients in collaborative care settings are likely to use apps, what features of mobile apps are the most beneficial in this setting, and what patient population(s) may be the most likely to benefit from app-augmented collaborative care [11]. For example, in the broader literature on mHealth, there is concern that older individuals may be less familiar with technology and may therefore be less inclined to engage with it [21]; however, this has not been systematically examined, to our knowledge, in the collaborative care setting. The limited existing studies suggest that overall, app usage among patients in collaborative care can be variable [9,12]. Understanding app usage is applicable to the optimization of mHealth platform interventions and their implementation in collaborative care [22]. If known, this information could lay the groundwork for improving the design and implementation of mobile apps in collaborative care.

### Objectives

This study describes a feasibility study of the Valera Health mobile platform and app (Valera Health Inc), which was used to augment collaborative care within primary care practices in a large health care system. Our primary aim was to assess the feasibility of app usage, which was measured based on engagement. A secondary outcome was preliminary clinical improvement in depression scores.

## Methods

### Ethics Approval

The study methods were approved by the Institutional Review Board of Northwell Health (approval number: 20-0545-NH). Informed consent was not sought due to the retrospective nature of the study.

### Study Overview

This was a retrospective review of a longitudinal, single-arm implementation initiative wherein individuals who were referred to the collaborative care program by their primary care providers (PCPs) were invited to participate in app-augmented collaborative care by the behavioral health care manager (BHCM). Individuals who were qualified to participate and agreed to do so were asked to download the Valera Health mobile app. Participants were told to use the app to complete in-app PHQ-9 measures, which were sent by the BHCM at preset monthly intervals; communicate with the BHCM through asynchronous chats as needed; and access the psychoeducational content in the app. Participants also experienced all usual collaborative care interventions, as described in the *Study Setting* section, including office visits and telephone contacts with the BHCM, short-term psychotherapy, care coordination, psychiatric case reviews, follow-ups with their PCPs as indicated, and the prescription of recommended psychiatric medications if indicated. PHQ-9 assessments were able to be completed through the app or on paper during office visits with the BHCM. For our primary outcome—feasibility—usage data on the number of user actions that were completed in the app were recorded by the app throughout the participation period. For our secondary outcome—clinical improvement—baseline and final PHQ-9 scores were compared.

### Study Setting

The study was conducted in a large, primarily suburban, academic health care system with multiple affiliated primary care practices. The primary care practices that were involved in this study provide behavioral health services through a system that was modeled after the CoCM introduced in the Improving Mood—Providing Access to Collaborative Treatment trial [6]. Briefly, in this model, patients presenting for routine primary care are systematically screened for depression by their PCPs using the PHQ-9—a tool that has been validated for this purpose [23]. Patients who screen positive on the instrument and/or, in the opinion of the PCP, display clinical features that are concerning for a behavioral health disorder are referred to a BHCM who is physically embedded in the clinic. The BHCM maintains a registry of patients, tracks outcomes via serial PHQ-9 assessments, provides time-limited psychotherapy, coordinates referrals to continued treatment and/or a higher level of care when necessary, and liaises with a psychiatrist who provides remote supervision to multiple BHCMs. Psychopharmacologic recommendations are relayed to the PCP, who remains the prescriber and clinician of record.

### Recruitment

Recruitment was planned in 2 phases. The first phase lasted from November 2018 to June 2019 and included 1 primary care practice with 1 BHCM. A total of 5 additional practices and 2 BHCMs were added in phase 2, which lasted from November 2019 to March 2020. There were no differences in procedures between the two phases except for the number of clinics and BHCMs involved. Patients who were referred to the collaborative care program were invited to participate in app-augmented collaborative care by the BHCM during initial appointments. During recruitment at the initial visits, the BHCM guided the participants through the process of downloading and using the app, answered any initial questions, and provided written instructions on the use of the app. After the initial visits, the BHCM was available by phone to troubleshoot the app as needed. Recruitment was halted in March 2020 when the social distancing measures that were required to prevent the spread of COVID-19 in New York resulted in the remote provision of ambulatory behavioral health services.

Individuals who declined to participate or were excluded received usual collaborative care, including primary care and behavioral health services. Those who agreed to participate received usual collaborative care, as described above, with the Valera Health mobile app as augmentation.

Individuals were included if they were adults with a diagnosis of depression or anxiety. Individuals were also excluded if they did not speak English or had severe mental illnesses, suicidal or violent ideation, or substance abuse disorders. Also excluded were children and individuals who required a referral to a higher level of care or continued treatment after the completion of the program.

**Figure 1:**
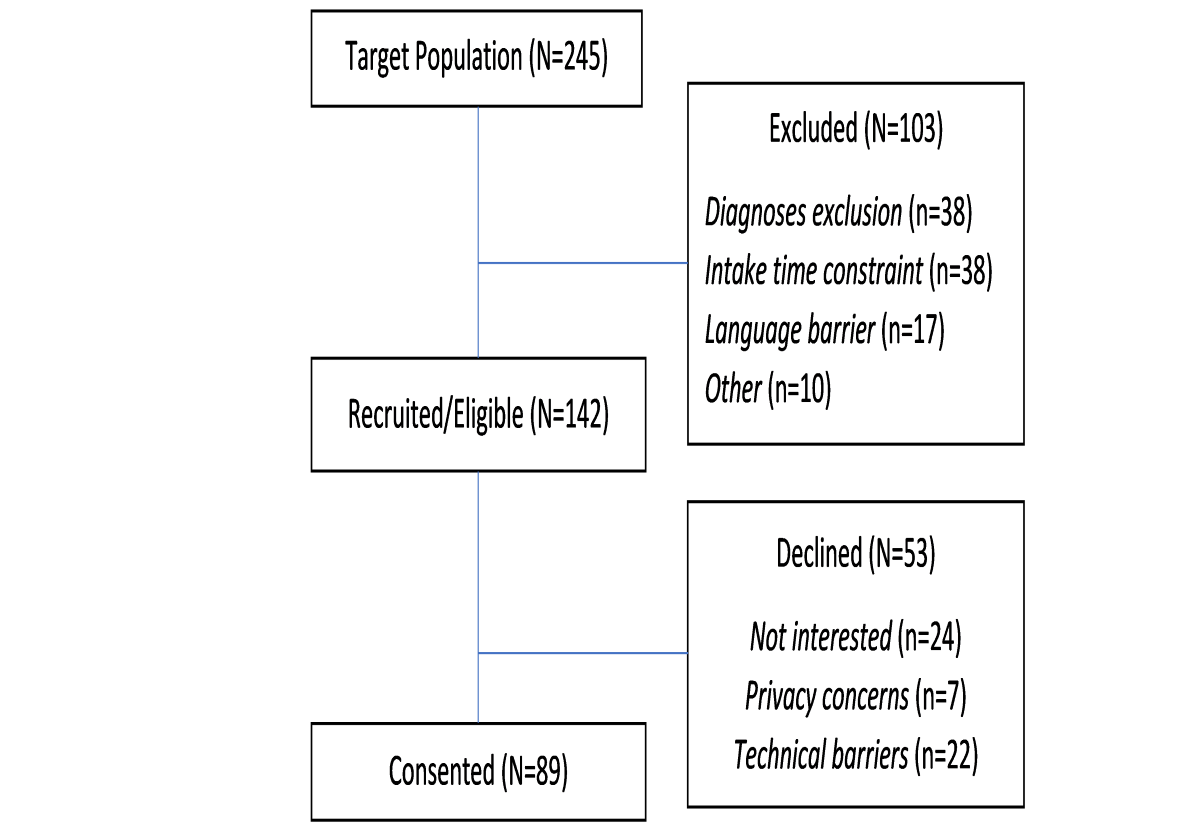
The flow of patient participation in the Valera Health mobile app pilot for behavioral health.

**Figure 2:**
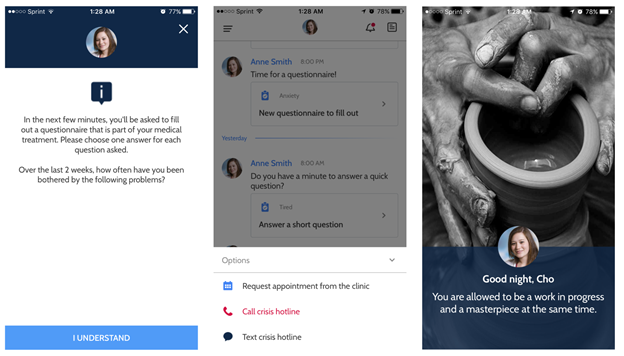
The patient user interface of and the experience on the Valera Health app.

Participation flow is illustrated by Figure 1. Between November 2019 and March 2020, a total of 245 individuals were referred by their PCPs to the collaborative care program for behavioral health services. Further, 58% (n=142) of these patients were eligible for and were recruited to participate in our study using the Valera Health mobile app (Figure 2), and 62.7% (89/142) of recruited patients consented to participate; 34 consented during the first phase of piloting the Valera Health mobile app, and 55 consented during the second phase. The time required to train patients in the use of the app was a barrier to recruitment among a sizable minority of individuals in the target population (38/245, 15.5%; Figure 1). In addition, a portion of our eligible patient population was unable to participate due to technical barriers (22/142,15.5%; Figure 1). Further, 7 individuals declined to participate due to privacy concerns (Figure 1). Participants were mostly female (60/89, 67%) and middle-aged (mean 38.6, SD 14 years). Participants were enrolled in the study for an average of 22 weeks.

### Intervention

The Valera Health mobile app is an English-language secure platform with several functionalities. Figure 2 shows screenshots of the app. First, the app automatically sends PHQ-9 assessments to participants at monthly intervals that are preset by the BHCM. Second, the app allows for secure asynchronous messaging between participants and the BHCM. BHCMs typically responded to chat messages from participants within 1 business day. The app also contains psychoeducational content. This content includes written material about common behavioral health conditions such as depression and anxiety, education about treatments like medication and psychotherapy, instructional guides on topics such as mindfulness, and video and audio clips on these topics. The BHCM had the option of prompting the participants to access psychoeducational content that was relevant to the participants’ care via the app. The Valera Health app was not integrated into the electronic health record (EHR). Relevant clinical information from the app, such as PHQ-9 scores, was documented into the EHR by the BHCM.

### Outcomes

We assessed the feasibility of the mHealth intervention by investigating engagement with the app and improvement in clinical outcomes. The primary outcome—the feasibility of engagement—was assessed based on app usage, which was measured as the number of participant-generated actions completed in the app. Possible participant-generated actions were (1) the in-app completion of PHQ-9 assessments, (2) the sending of a chat message, or (3) the accessing of in-app psychoeducational material. App usage was monitored throughout the study by the Valera Health app. Engagement was stratified by age and sex. A secondary outcome was clinical improvement, which we defined as a final PHQ-9 score of less than 10 or a greater than 50% reduction in PHQ-9 scores. Baseline PHQ-9 scores were assessed during intake by the BHCM. The final PHQ-9 scores were the last ones recorded for the participants and were extracted from the EHR. Any PHQ-9 assessment, whether it was completed through the app or on paper during office visits with the BHCM, was considered in the analysis of clinical improvement.

### Data Analysis

Data were analyzed by using Microsoft Excel, and an α of .05 was set as the a priori level of significance. Simple descriptive statistics of app usage were used to report on feasibility and engagement metrics. A Kruskal-Wallis equality-of-populations rank test of median differences in total mobile app actions was used to analyze differences in app usage between age and sex groups. For our secondary outcome—clinical improvement—a 2-tailed, 2-sample Welch *t* test with unequal variances was used to analyze the difference between baseline and follow-up PHQ-9 scores.

## Results

Table 1 describes the brief demographic and engagement characteristics of participants. Only 12% (11/89) never engaged with the app during the trial. Across all participants, we observed a median engagement of 7 (IQR 12; mean 10.4; range 0-130) actions in the app. At least 1 action in the mobile app was completed by 87% (78/89) of participants. Psychoeducational content was reviewed by 75% (67/89) of participants (number of articles reviewed: median 2, IQR 4; range 0-18). Chat messages were sent by 62% (55/89) of participants. Participants sent a median of 1 (IQR 5; range 0-115) chat message used for either scheduling an appointment or reporting symptoms.

A baseline PHQ-9 score was reported for 97% (86/89) of study participants. However, follow-up PHQ-9 assessments were available only for 49% (44/89) of participants. PHQ-9 scores improved from baseline to follow-up for 73% (32/44) of participants for whom we had baseline and follow-up PHQ-9 scores (n=44). The percentages of participants with improved PHQ-9 scores were not different by sex (*P*=.53) or age groups (18-35, 36-55, and ≥56 years: *P*=.90), although as previously noted, the sample of participants with recorded follow-up PHQ-9 scores was notably smaller than the sample of participants with baseline PHQ-9 scores.

**Table 1:** Demographic and engagement characteristics of behavioral health patients who participated in the Valera Health mobile app study (N=89).

		Value, n (%)	Age (years), mean (SD)	Baseline PHQ-9^a^ score, mean (SD)^b^	Follow-up PHQ-9 score, mean (SD)^c^	*P* value^d^	Total mobile app actions, median (IQR)	*P* value^e^	
Total		89 (100)	38.6 (14.5)	11.5 (5.3)	8.6 (4.6)	.002	7 (12)	N/A^f^	
**Sex**									.84
	Female	60 (67)	38.4 (14.6)	11.5 (5.3)	9.0 (4.8)	.03	6.5 (12)		
	Male	29 (33)	39.1 (14.6)	11.7 (5.5)	7.5 (3.9)	.01	8 (11)		
**Age (years)**									.42
	18-35	44 (51^g^)	N/A	11.8 (5.1)	8.7 (4.0)	.01	8 (14)		
	36-55	31 (36^g^)	N/A	11.1 (4.9)	8.3 (5.9)	.13	6 (9)		
	>55	12 (14^g^)	N/A	11.7 (7.2)	8.9 (4.3)	.29	5.5 (11.5)		

^a^PHQ-9: Patient Health Questionnaire-9.

^b^n=86.

^c^n=44.

^d^A 2-sample Welch *t* test with unequal variances between baseline and follow-up PHQ-9 scores.

^e^A Kruskal-Wallis equality-of-populations rank test of median differences in the total mobile app actions.

^f^N/A: not applicable.

^g^The denominator for this percentage is 87.

## Discussion

### Principal Findings

This study reports on the evaluation of one of the first implementations of mobile app–augmented care within a collaborative care program. The overarching purpose of this feasibility study was to understand whether patients in collaborative care are likely to participate in app-augmented care, what features of the app are used, and whether demographic differences exist among app users in this context. Overall, our results indicate the acceptability and feasibility of app usage; the overwhelming majority of participants (78/89, 87%) used the app at least once, and a modest median of 7 actions were completed in the app. Encouragingly, all of the features of the app were used at similar rates. In particular, the psychoeducational materials and chat feature were both popular functions, suggesting that the app may indeed act to reinforce the concepts that are learned during clinical encounters and can enhance communication between patients and care providers as postulated in the literature [13]. There was no significant difference in app usage by age group (*P*=.42). Finally, we were encouraged by the finding that privacy—a concern in the broader literature surrounding app usage—was an infrequent cause for declining to participate (n=7) in our study, further suggesting acceptability [24,25].

With regard to our secondary outcome, the preliminary clinical outcomes of app-augmented care in this study were encouraging, with 73% (32/44) of participants for whom follow-up PHQ-9 data were available experiencing improvements (n=44). Our findings on clinical outcomes are limited by the considerable drop-off in the number of participants who completed a follow-up PHQ-9 assessment. The reasons for such decreases are not completely known and include app attrition, which is consistent with previous literature showing that attrition is a challenge to the implementation of mobile technologies [12,26]. The missing data also introduced bias into the study, as the population of participants who did not complete follow-up assessments may not be random.

### Implications

Our findings reveal several issues that deserve consideration and optimization prior to subsequent implementation efforts. First, despite the prevalence of smartphones, a nontrivial portion of our eligible patient population was unable to participate due to technical barriers (22/142, 15.5%). In addition, the time required to train patients in the use of the app was a barrier to recruitment among a sizable minority of individuals in the target population (38/245, 15.5%). To address these issues, previous literature has postulated the need for a digital health navigator—a new team member with expertise in digital and mobile strategies who can help educate patients on the use of these tools, thereby reducing the burden among staff who may lack this expertise and have insufficient time to address these topics during appointments [14,27,28]. Digital health navigators can be instrumental to training staff in rapidly developing competencies for mHealth [29]. The CoCM, which already operates in a framework of interdisciplinary collaboration, may be uniquely suited to the adoption of the digital health navigator role in the future.

Second, future implementation efforts should carefully consider measures for mitigating the potential unintended negative consequences of app use. For example, the chat function could give participants the perception of continuous access to the clinician. Though most participants in our study used the chat a moderate amount of times, there was substantial variability, with some participants sending more than 100 messages. Care provider burnout is a concern in the face of such significant increases in patient communication. The future implementation of this and similar technologies would benefit from integration with existing health information systems and care provider workflows to better support care provider decision-making.

Finally, while our results indicate preliminary evidence that patients are willing to participate in app-augmented collaborative care, there exist many opportunities for the optimization of engagement. For example, our intervention allowed the BHCM to nudge patients to engage with psychoeducational materials. Higher engagement can potentially be achieved by personalizing technology to deliver the right content in the right amount and at the right time [11,30,31]. Future research should identify pathways for personalization and investigate its effects on engagement and clinical improvement.

### Limitations

Our study has several limitations. First, qualitative data for contextualizing findings were not systematically collected as a part of this study. Second, the experiences of the BHCMs were also not systematically evaluated. This study examined app usage among participants, which was stratified by age and sex; however, other demographic factors of potential interest, such as race, ethnicity, insurance status, were not tracked. Finally, this was a feasibility study; thus, conclusions are limited by the lack of a control group and the unknown characteristics of nonparticipants. Future research should employ a rigorous clinical trial involving patient and/or clinic randomization to evaluate clinical effectiveness.

### Conclusions

In conclusion, our preliminary findings indicate the moderate feasibility of using mHealth technology to augment behavioral health care in primary care settings. The results of this study are applicable to improving the design and implementation of mobile apps in collaborative care.

## Abbreviations

BHCM: behavioral health care manager
CoCM: collaborative care model
EHR: electronic health record
mHealth: mobile health
PCP: primary care provider
PHQ-9: Patient Health Questionnaire-9
